# Syk Signaling in Dendritic Cells Orchestrates Innate Resistance to Systemic Fungal Infection

**DOI:** 10.1371/journal.ppat.1004276

**Published:** 2014-07-17

**Authors:** Paul G. Whitney, Eva Bär, Fabiola Osorio, Neil C. Rogers, Barbara U. Schraml, Safia Deddouche, Salomé LeibundGut-Landmann, Caetano Reis e Sousa

**Affiliations:** 1 Immunobiology Laboratory, Cancer Research UK, London Research Institute, Lincoln's Inn Fields Laboratories, London, United Kingdom; 2 Institute of Microbiology, ETH Zurich, Zürich, Switzerland; University of Pittsburgh, United States of America

## Abstract

Host protection from fungal infection is thought to ensue in part from the activity of Syk-coupled C-type lectin receptors and MyD88-coupled toll-like receptors in myeloid cells, including neutrophils, macrophages and dendritic cells (DCs). Given the multitude of cell types and receptors involved, elimination of a single pathway for fungal recognition in a cell type such as DCs, primarily known for their ability to prime T cell responses, would be expected to have little effect on innate resistance to fungal infection. Here we report that this is surprisingly not the case and that selective loss of Syk but not MyD88 in DCs abrogates innate resistance to acute systemic *Candida albicans* infection in mice. We show that Syk expression by DCs is necessary for IL-23p19 production in response to *C. albicans*, which is essential to transiently induce GM-CSF secretion by NK cells that are recruited to the site of fungal replication. NK cell-derived-GM-CSF in turn sustains the anti-microbial activity of neutrophils, the main fungicidal effectors. Thus, the activity of a single kinase in a single myeloid cell type orchestrates a complex series of molecular and cellular events that underlies innate resistance to fungal sepsis.

## Author Summary

Multiple cell types bearing a vast array of immune receptors with different modes of signaling ensure that the host response to infection is both robust and reliable. For this reason, loss of a single signaling pathway in a given cell type is often not enough to impact host resistance. Here, we find, surprisingly, that this is not the case in a mouse model of systemic fungal infection with *Candida albicans*. We show that a single kinase (Syk) in a single cell type (dendritic cells, DCs) coordinates the entire host resistance network. We highlight Syk-dependent production of IL-23p19 by DCs as the key to protection and show that IL-23p19 acts on another white blood cell type, NK cells, to specifically induce production of another mediator, GM-CSF. The latter is key for yet another cell, the neutrophil, to be mobilized into action and kill *Candida* organisms. This study places DCs, best known for their role in priming T cells, at the center of a cellular relay of innate immunity to fungal infection. It highlights key nodes of antifungal immunity that could be targeted in combination with antifungal drugs to provide new ways to treat patients with fungal sepsis, who generally have poor outcomes.

## Introduction

*Candida albicans* is the most prevalent fungal pathogen in humans causing local infections of skin, nails, oral cavity and genital tract [1]. In some instances, *Candida* can spread systemically via the bloodstream and lodge in the kidneys, which then act as the major site of fungal replication [2]. Despite the availability of several anti-fungal drugs, invasive candidiasis still has a high mortality rate ranging from 45 to 75% [3], highlighting the need to further understand host-pathogen interactions and mechanisms of immune resistance to fungal spread.

Despite its potential pathogenicity, *C. albicans* generally behaves as an innocuous commensal in immunocompetent individuals because it triggers host defense pathways that keep the organism in check. Host protection from infection ultimately depends on recognition of *Candida* by pattern recognition receptors (PRRs) and their associated signaling pathways that initiate immunity. Many PRRs recognizing *Candida* are expressed by myeloid cells and belong either to the Toll-like receptor (TLR) or the C-type lectin receptor (CLR) families. A role for TLRs in anti-fungal defense was first suggested by studies in mice deficient for the TLR adaptor MyD88, which are highly susceptible to systemic candidiasis [4], [5]. However, MyD88 additionally transduces signals from IL-1 and IL-18 receptors, which can impact innate anti-fungal immunity [4], [6]–[10], and human deficiency in MyD88 does not lead to loss of resistance to fungal organisms [11]. Therefore, the role of TLRs in protection from *Candida* infection remains unresolved [12]–[14].

In contrast, the role of CLRs in anti-fungal defense is becoming increasingly well-established. CLRs involved in fungal recognition include Dectin-1, Dectin-2, mannose receptor, MCL and Mincle, and mice or humans deficient in some of these receptors display enhanced susceptibility to candidiasis [15]–[19]. Dectin-1, -2 and Mincle all signal via tyrosine-based motifs that recruit the spleen tyrosine kinase Syk [20]–[23], leading to an NF-κB-dependent transcriptional program via CARD9 [24]. CLR/Syk signaling additionally promotes activation of NFAT, MAP kinase and PI3 kinase (PI3K) pathways [25], [26] and can also lead to production of reactive oxygen species (ROS) and activation of inflammasomes [6]. Notably, Syk- or CARD9-deficient dendritic cells (DCs) fail to produce certain cytokines in response to *Candida* and fungal cell wall components [6], [21], [27] and CARD9-deficient mice are highly susceptible to systemic infection with *C. albicans* [24]. Likewise, human deficiency in CARD9 results in severe forms of superficial as well as invasive candidiasis [28], [29]. Thus, Syk-dependent signaling by CLRs appears an important and non-redundant pathway for anti-fungal responses. It is presently unclear whether this reflects a dominant role for Syk in a given myeloid cell type or the additive effects of PRR signaling in multiple phagocytes.

PRR signaling can trigger both innate and adaptive immune mechanisms. Adaptive immunity is initiated by DCs and important for defense against mucocutaneous candidiasis [30] but does not play a prominent role in combatting disseminated *C. albicans* infection [31]. Instead, innate immunity acts as the major barrier to systemic *Candida* spread. Indeed, the candidacidal activity of neutrophils is the key mediator of immunity to systemic candidiasis and neutropenia is a major risk factor for invasive *Candida* disease [31], [32]. Macrophages and inflammatory monocytes also coordinate aspects of resistance to systemic *Candida* spread [33]–[36] while, recently, NK cells have been shown to be crucial for promoting neutrophil candidacidal activity during experimental systemic candidiasis in mice [37]. The collaborative impact of NK cells and neutrophils is also apparent in a model of invasive *Aspergillus fumigatus* where co-depletion greatly decreases survival compared to neutrophil depletion alone [38]. Thus, neutrophils, monocytes/macrophages and NK cells all mediate innate resistance to fungal hematogenous spread although whether all these cell types act individually or coordinately to provide host protection and which signals are involved in regulating their activity remains unknown.

Experimental systemic candidiasis in mice mimics human candidemia in that fungal replication occurs primarily in the kidneys and resistance is mediated by neutrophils independently of T and B cells [39]. In this work, we report that the coordination of innate immunity to systemic *C. albicans* infection in mice is critically dependent on Syk and not MyD88 expression in CD11c^+^ cells. We identify the CD11c^+^ cells in question as DCs by ontogenetic criteria, thereby ascribing DCs a key role in innate immunity that is much less appreciated than their function in adaptive immunity. We show that this is because in the absence of Syk signaling, DCs do not produce IL-23p19 in response to *C. albicans*, which is necessary for fungus-driven production of GM-CSF by NK cells in the kidney. The loss of GM-CSF-producing NK cells leads to a failure to sustain the candidacidal ability of neutrophils and results in high kidney fungal burden and decreased survival to infection. Thus, effective immunity to systemic *C. albicans* infection involves a precise chain of sequential cellular activation events that is initiated by Syk-dependent signaling in DCs, depends on NK cells and culminates in neutrophil fungicidal activity.

## Results

### Increased susceptibility of CD11cΔSyk mice to systemic *C. albicans* infection

To assess the relative contribution of Syk- and MyD88-dependent pathways in CD11c^+^ mononuclear phagocytes (predominantly DCs) to immunity during systemic *C. albicans* infection, we crossed Syk^fl/fl^ [40] or MyD88^fl/fl^ [41] strains to CD11c-Cre [42] mice to generate CD11cΔSyk and CD11cΔMyD88 lines, respectively. When CD11c^+^ MHC II^+^ cells (henceforth called DCs – see below) in the spleens and kidneys of CD11cΔSyk mice were compared to those in littermate controls (CD11cCre^−^ Syk^fl/fl^), DCs from CD11cΔSyk mice displayed a marked reduction in Syk mRNA, with a PCR signal barely above that obtained for T cells, which do not express the kinase (Figure S1A). No reduction in Syk mRNA was seen in B cells (Figure S1A) and measurement of Syk protein levels by intracellular staining (Figure S1B and S1C) or Western blotting (data not shown) confirmed that the kinase was specifically deleted in CD11c^+^ cells. Importantly, levels of Syk were not reduced in neutrophils, indicating restriction of the deletion to the mononuclear phagocyte system (Figure S1B and S1C). Likewise, in CD11cΔMyD88 mice, a reduction in MyD88 staining was observed specifically in CD11c^+^ MHC-II^+^ DCs and not in neutrophils or other leukocytes (Figure S1D and data not shown), as reported [41].

**Figure 1:**
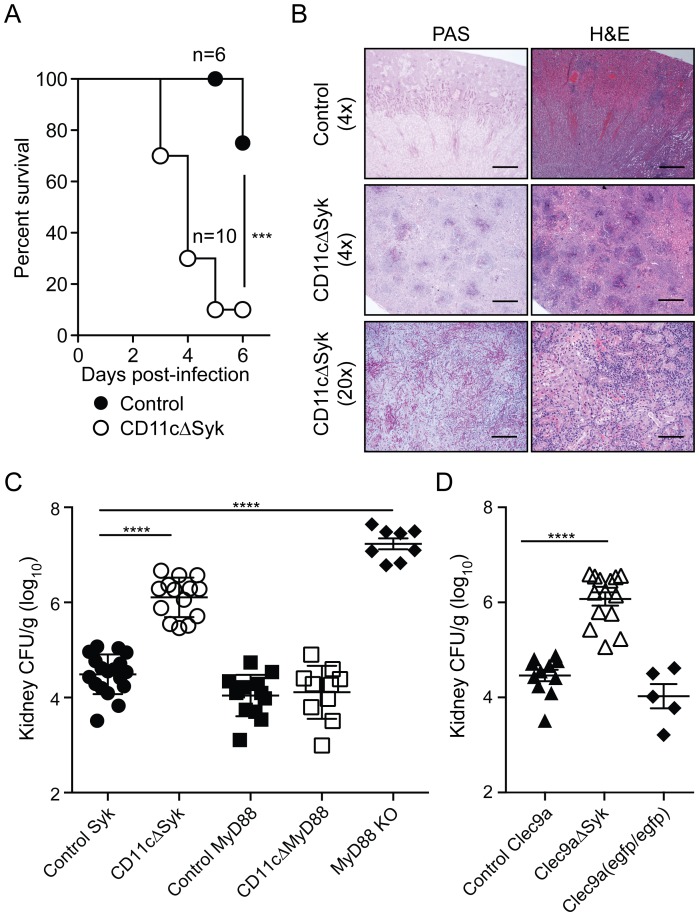
Mice with targeted deletion of Syk in CD11c^+^ cells show increased susceptibility to systemic candidiasis. (A) Control and CD11cΔSyk mice were infected with 5×10^4^ CFU of *C. albicans* intravenously. Survival data are presented as a Kaplan-Meier plot with a log rank test used to compare susceptibility between the two groups. Data are pooled from two independent experiments. (B) 4× and 20× magnification of Periodic Acid Schiff (PAS) or Hematoxilyn and Eosin (H&E) stained kidney sections collected at the endpoint of the experiment shown in (A). Scale bars are 50 µm (4× magnification) and 250 µm (20× magnification), respectively. (C–D) control Syk, CD11cΔSyk, control MyD88, CD11cΔMyD88 and MyD88 KO mice (C) and control Clec9a, Clec9aΔSyk and Clec9a(egfp/egfp) mice (D) were infected with 2×10^5^ CFU of *C. albicans* intravenously. Kidneys were removed 2 days post-infection and analyzed for fungal burden. CFU are calculated per gram of kidney. Data shown are mean +/− SEM from pooled from two to six independent experiments with each symbol representing an individual mouse with statistical significance of any differences determined using a 1-way ANOVA with Tukey post-test analysis.

Remarkably, CD11cΔSyk mice succumbed rapidly to systemic infection with 5×10^4^ CFU of *C. albicans* when compared to controls, of which the majority survived for up to 3 weeks (Figure 1A and data not shown). At higher inoculum doses, the mortality of control animals increased [43], although, importantly, the difference in susceptibility between control and CD11cΔSyk mice was maintained (data not shown). The kidneys of infected CD11cΔSyk mice showed a large number of fungal abscesses with prominent hyphae (revealed by periodic acid Schiff (PAS) staining) heavily surrounded by leukocytes (shown by hematoxylin and eosin (H&E) staining) (Figure 1B). Consistent with these observations, fungal burden in the kidneys of CD11cΔSyk mice was around 100-fold higher than in control littermates (Figure 1C). Reflecting the massive candidemia, fungus could additionally be recovered from spleen and liver of CD11cΔSyk mice, which additionally displayed liver lipolysis (data not shown). In contrast, selective ablation of MyD88 in CD11c^+^ cells in CD11cΔMyD88 mice did not result in enhanced susceptibility to systemic *Candida* infection even though MyD88-deficient mice (lacking MyD88 in all cell types) were extremely susceptible (Figure 1C). Thus, ablation of Syk but not MyD88 in CD11c-expressing cells greatly compromises innate resistance to systemic *C. albicans* infection in mice and leads to death from fulminant candidiasis.

CD11c is not an exclusive marker of DCs. To narrow down the CD11c^+^ cell type required to express Syk in this model, we made use of recently-developed Clec9a-Cre mice in which Cre activity is restricted to cells derived from non-monocytic conventional DC precursors (CDP) [44]. Notably, despite the incomplete penetrance of Cre-mediated recombination in such precursors [44], Clec9aΔSyk were nearly as susceptible as CD11cΔSyk mice to systemic *Candida* infection (Figure 1D and data not shown). Because Clec9aΔSyk mice were used as homozygotes in these experiments and therefore lacked DNGR-1 expression [44], we confirmed that DNGR-1 deficiency does not impact on susceptibility to candidiasis by assessing fungal burden in infected Clec9a^egfp/egfp^ mice [45] (Figure 1D). These data therefore suggest a key role for Syk signaling by conventional DCs. Consistent with that conclusion, all kidney DC sub-populations in CD11cΔSyk mice showed loss of Syk independently of infection (Figure S1F). We conclude that Syk expression by DCs and, possibly, additional CD11c^+^ cells is a key determinant of innate immunity to systemic *C. albicans* infection.

### Defective neutrophils in the kidneys of candida-infected CD11cΔSyk mice

We assessed the composition of the leukocytic infiltrate in kidneys of infected CD11cΔSyk mice to determine if susceptibility to candidiasis correlated with loss of any particular CD11c^+^ phagocyte subset whose development or recruitment to the site of infection might depend on Syk. Interestingly, there was little change in the total size of the CD11c^+^ MHC-II^+^ DC compartment after infection (Figure S2A), although its relative composition was altered: in kidneys from uninfected mice, CD11b^INT^ F4/80^+^ DCs were prominent whilst in infected mice this population decreased in size and a CD11b^+^ F4/80^INT^ population became more abundant (Figure S2B and S2C). Importantly, despite infection-induced changes, there was no difference between control or CD11cΔSyk mice. For example, the total number of CD11c^+^ MHC-II^+^ cells was the same in the two strains and there was only a marginal difference in percentage (Figure S2A). Similarly, the change in hierarchy of CD11c^+^ MHC-II^+^ populations following infection was largely equivalent between the strains (Figure S2C). Small differences observed for the percentage but not total number of CD11c^+^ MHC-II^+^ CD11b^INT^ F4/80^+^ cells (Figure S2C) might reflect changes in other leukocyte populations even though there was no obvious change in B, T or NK cells (data not shown).

**Figure 2:**
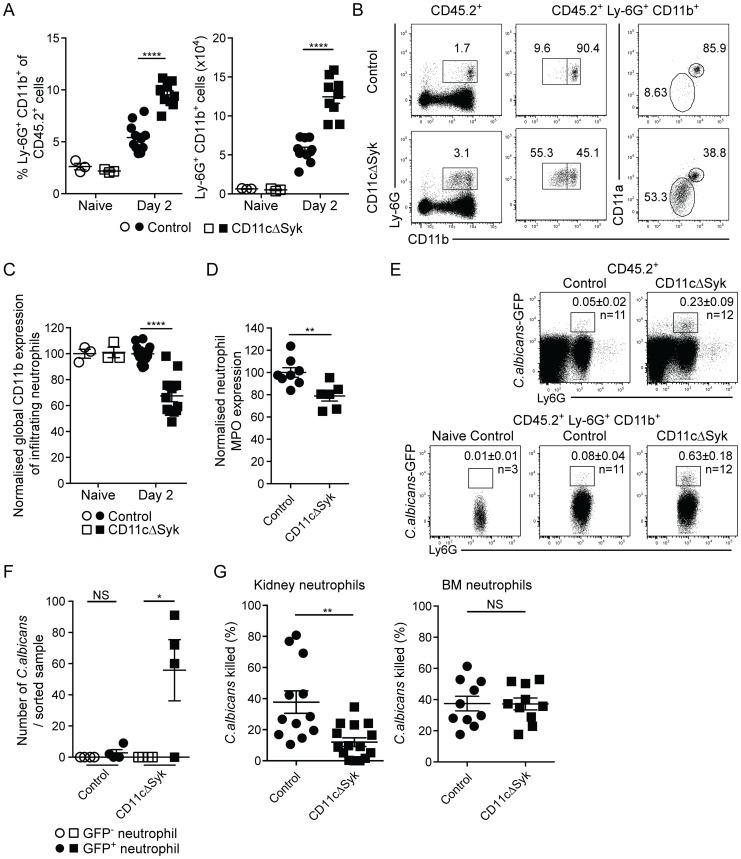
Increased infiltration of defective neutrophils in the kidneys of CD11cΔSyk mice. Control and CD11cΔSyk mice were infected with 2×10^5^ CFU of *C. albicans* intravenously. Kidneys were removed from naïve mice or mice 2 days post-infection and leukocytes were surface stained for CD11b, F4/80, MHC-II and Ly-6G. (A) Percentage and total number of Ly6G^+^ CD11b^+^ kidney neutrophils. Data are mean +/− SEM from four pooled independent experiments with each data point representing an individual mouse. (B) Representative expression of CD11b and CD11a on infiltrating kidney neutrophils of day 2 infected control and CD11cΔSyk mice. (C) Global geometric mean of CD11b expression on infiltrating kidney neutrophils normalized against control naïve mice. Data are mean +/− SEM from four independent experiments with each data point representing an individual mouse. (D) Neutrophils from day 2 infected mice were permeabilized and stained for MPO. Data shown are global geometric mean of MPO signal on infiltrating kidney neutrophils normalized against control mice. Data are combined from two independent experiments with each data point representing an individual mouse. (E) Control and CD11cΔSyk were infected with 2×10^5^ CFU of *C. albicans*-GFP intravenously. Kidneys were removed 2 days post-infection and analyzed for GFP expression. Representative staining profiles of total CD45.2^+^ kidney cells (top panel) and CD45.2^+^ Ly-6G^+^ neutrophils (bottom panel). Boxes indicate the percentages of GFP^+^ neutrophils and the mean +/− SEM from three independent experiments with number (n) of mice indicated. (F) GFP^+^ and GFP^−^ neutrophils (CD11b^+^ Ly-6G^+^ CD11c^−^ F4/80^−^) were sorted, lysed in water and plated to determining presence of viable fungi. Data are combined from two independent experiments with each data point representing cells sorted from an individual mouse. (G) Neutrophils were sorted from naïve BM or day 2 infected kidneys of control and CD11cΔSyk mice and incubated with *C. albicans* (10∶1) for 1 h at 37°C. The survival of fungi was then assessed. Data are mean +/− SEM from three independent experiments with each data point representing cells sorted from an individual mouse. Statistical significance of any differences for A, C, D and G was determined by 2-tailed *t* test. Whilst a Kruskal-Wallis with Dunn's multiple comparison test was undertaken for F. NS, not significant.

In contrast to the CD11c^+^ mononuclear phagocyte pool, the numbers and percentages of CD11c^−^ MHC-II^−^ neutrophils increased greatly in the kidneys following infection in both strains (Figure 2A), as expected [43], [46]. However, CD11cΔSyk mice displayed higher levels of kidney neutrophilia, correlating with the greater fungal burden (Figure 2A). Importantly, the phenotype of neutrophils in the kidneys but not the bone marrow of infected CD11cΔSyk mice was atypical, with a large fraction of the cells expressing only low levels of CD11b and CD11a (Figure 2B, 2C and data not shown). The cells were also less granular but did not appear apoptotic or stain for active caspase 3 (data not shown). We further assessed levels of myeloperoxidase (MPO), a major constituent of azurophil granules necessary for generation of reactive oxygen species (ROS), a key component of the neutrophil killing arsenal [47]. Kidney neutrophils from infected CD11cΔSyk mice had decreased levels of MPO when compared to controls (Figure 2D). As these phenotypic differences might suggest impaired functionality [48], we assessed the ability of neutrophils in CD11cΔSyk mice to kill *C. albicans*. We infected control and CD11cΔSyk mice with a strain of GFP-expressing *C. albicans* and measured GFP signal among kidney leukocyte populations. As expected, the majority of the GFP signal was found within neutrophils (Figure 2E). However, a greater frequency of GFP^+^ neutrophils were present in CD11cΔSyk mice than in controls suggesting that kidney neutrophils from the former strain are impaired in their ability to destroy the fungus. To explicitly test this hypothesis, we sorted GFP^+^ and GFP^−^ neutrophils from the kidney, lysed them and plated the lysates to determine *C. albicans* growth. This analysis confirmed that GFP^+^ neutrophils derived from CD11cΔSyk but not from control mice contained live *C. albicans* (Figure 2F). We then evaluated if neutrophils could kill *C. albicans ex vivo* by sorting GFP^−^ neutrophils and incubating them with live fungus. Consistent with their phenotypic differences, neutrophils from kidneys of infected CD11cΔSyk mice showed a decreased ability to kill *C. albicans ex vivo* when compared to their counterparts from control infected mice (Figure 2G). In contrast, bone marrow neutrophils from either uninfected or infected CD11cΔSyk mice showed equivalent *ex vivo* candidacidal capacity (Figure 2G and data not shown), which argues that neutrophil impairment occurs locally at the site of infection. We conclude that in CD11cΔSyk mice infected systemically with *C. albicans* there is undiminished recruitment of neutrophils to the kidney but the recruited cells display phenotypic alterations and are locally impaired in their candidacidal activity.

### Loss of GM-CSF in kidney underlies the susceptibility of CD11cΔSyk mice to systemic *Candida* infection

**Figure 3:**
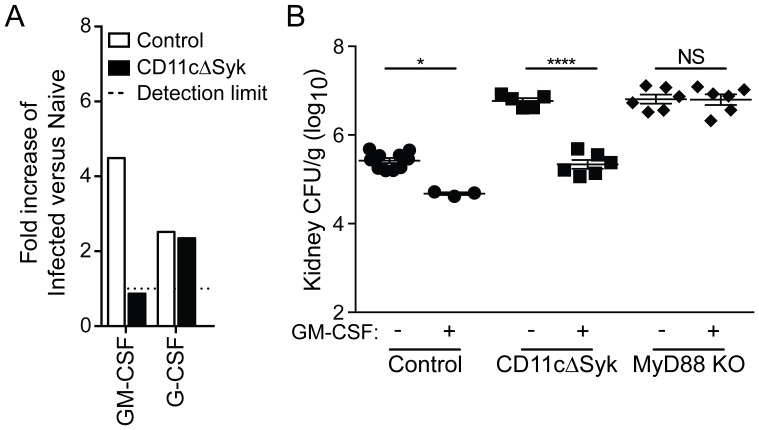
Selective loss of GM-CSF in the kidneys of CD11cΔSyk mice and restoration of fungal control by exogenous GM-CSF administration. (A) Kidneys were removed from naïve mice or mice 16 h post-infection mice and assessed using a Proteome profiler. Data show relative pixel density of duplicate blots for GM-CSF and G-CSF protein expression changes following infection. (B) Control, CD11cΔSyk and MyD88 KO mice were given PBS or GM-CSF at the time of *C. albicans* infection as indicated and a second dose 24 h later. Fungal burden was assessed 2 days post-infection. Data are mean +/− SEM from two pooled experiments with each symbol representing an individual mouse. Statistical significance of any differences was determined by 2-tailed *t* test. NS, not significant.

We searched for local alterations in the inflammatory milieu of the kidney that might connect diminished neutrophil function to loss of Syk in DCs. Homogenates of kidneys from CD11cΔSyk mice showed higher levels of IL-6, KC, MIP-1α, IL-1β, TNF, IL-1α and MCP-3 at days 1 or 2 post-infection when compared to control mice (Figure S3A and S3B). This likely reflects the contribution of cell types other than DCs and macrophages as some of those cytokines are known to be produced in a Syk-dependent manner by mononuclear phagocytes in response to stimulation with *Candida albicans* [6], [27], [49]. Because the interpretation of the data was marred by the large differences in fungal burden between the two strains, a broader analysis was performed early after infection (16 h) when fungal burdens are more equivalent. This analysis confirmed the discrepancy in IL-6 levels between infected mouse strains while revealing that many inflammatory mediators are in fact induced to similar levels in both control and CD11cΔSyk infected mice (Figure S3C). A notable exception is GM-CSF, which was found to be selectively lost in the kidneys of infected CD11cΔSyk mice when compared to controls (Figure 3A).

GM-CSF has been reported to be important for enhancement of neutrophil maturation and neutrophil oxidative responses in both mice and man [50]–[52]. We therefore tested whether exogenous GM-CSF could decrease the susceptibility of CD11cΔSyk mice to infection. Recombinant GM-CSF administration resulted in a marked decrease in fungal burden in the kidneys of infected CD11cΔSyk mice (Figure 3B). In contrast, the same GM-CSF treatment had only a modest effect in control mice and, importantly, did not impact the hyper-susceptibility of MyD88 KO mice, demonstrating selectivity (Figure 3B). Altogether, these data suggest that the susceptibility of CD11cΔSyk mice to systemic *Candida* infection stems from a deficiency in GM-CSF production in the kidney, which results in failure to locally sustain neutrophil microbicidal activity.

### Impaired fungal control in CD11cΔSyk mice is due to a loss of GM-CSF-production by NK cells

**Figure 4:**
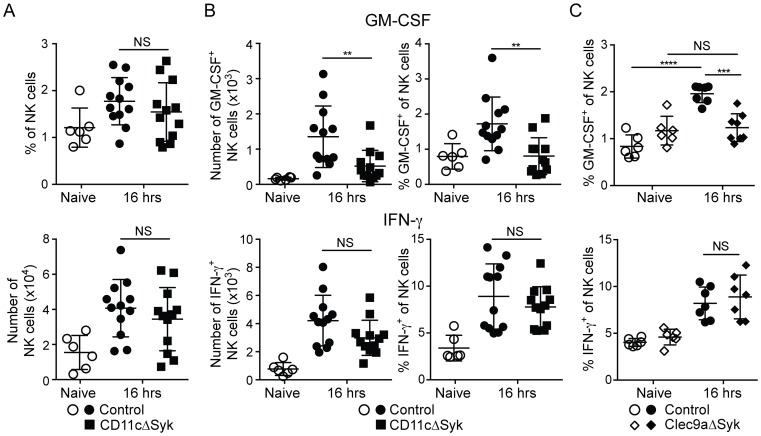
GM-CSF-producing NK cells are selectively reduced in infected CD11cΔSyk and Clec9aΔSyk mice. Control, CD11cΔSyk and Clec9aΔSyk mice were infected with 2×10^5^ CFU of *C. albicans* intravenously. (A) Percentage and total number of NK cells in the kidney of control and CD11cΔSyk mice that were either uninfected or infected 16 h earlier. (B–C) Enriched leukocyte populations from control and CD11cΔSyk mice (B) and from control and Clec9aΔSyk mice (C) that were either uninfected or infected 16 h earlier were analyzed for GM-CSF and IFN-γ by intracellular staining. The percentage and absolute number of cytokine-positive CD45.2^+^ NK1.1^+^ CD3^−^ cells are shown. Each data point represents an individual mouse with bars indicating mean +/− SEM pooled from two to four independent experiments. Statistical significance of any differences was determined using a 1-way ANOVA with Tukey post-test analysis. NS, not significant.

We have recently found that kidney-infiltrating NK cells serve as a non-redundant source of GM-CSF to promote the candidacidal activity of neutrophils during systemic *Candida* infection [37]. Therefore, we assessed recruitment of NK cells to the kidneys of infected control and CD11cΔSyk mice and measured their production of GM-CSF and IFN-γ. There was no difference between the two strains in the percentage or the total number of kidney NK cells either before or at different times after infection (Figure 4A and data not shown). However, as early as 16 h after infection, a marked reduction was observed in CD11cΔSyk mice in both the percentage and number of GM-CSF-producing NK cells (Figure 4B). In contrast, the percentage and number of NK cells positive for IFN-γ was equivalent between the two strains (Figure 4B). The production of GM-CSF by NK cells was transient as levels of the cytokine diminished after 16 h in contrast to those of IFN-γ, which continued to greatly increase (data not shown). A similar loss of GM-CSF^+^ but not of IFN-γ^+^ NK cells was seen in infected Clec9aΔSyk mice (Figure 4C), strengthening the notion that the phenotype stems from loss of Syk in DCs.

**Figure 5:**
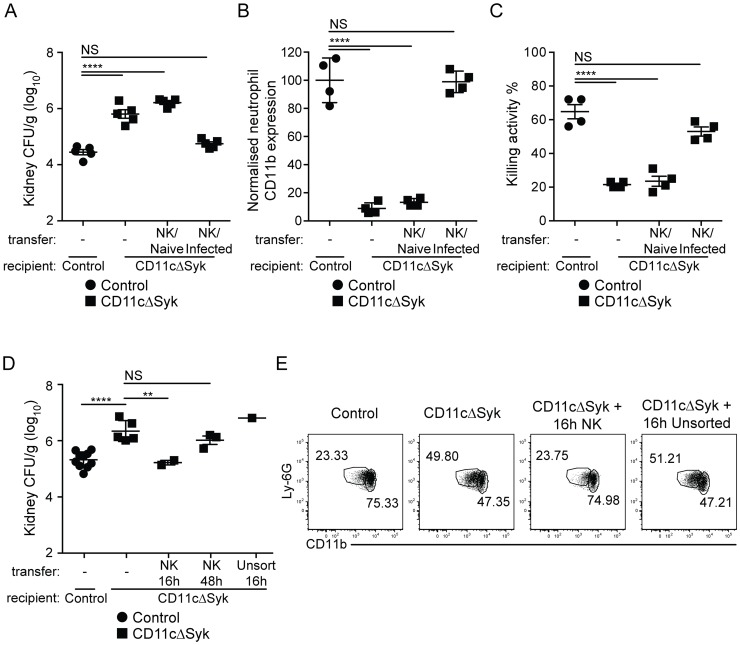
Transfer of *in vivo* activated NK cells leads to restoration of fungal control in CD11cΔSyk mice. (A) Fungal burden in kidneys from control and CD11cΔSyk mice, as well as CD11cΔSyk mice receiving adoptively transferred NK cell-enriched splenocytes from either naïve or day 1 infected wild type mice. Data are mean +/− SEM pooled from two independent experiments with each symbol representing an individual mouse. (B) Global geometric mean of CD11b expression by infiltrating neutrophils from the mice shown in (A). Data are mean +/− SEM from two independent experiments with each symbol representing neutrophils from an individual mouse. (C) Neutrophils were isolated from the blood of the mice shown in (A) and co-cultured with *C. albicans* to assess candidacidal activity. Data are mean +/− SEM from two independent experiments with each symbol representing an individual mouse. (D) Fungal burden of kidneys from control and CD11cΔSyk mice as well as CD11cΔSyk mice that received sorted splenic NK cells or unsorted total splenocytes isolated from 16 h or 48 h infected control mice. Data are mean +/− SEM from two independent experiments with each symbol representing an individual mouse. (E) Neutrophils identified as live, CD45.2^+^, Ly-6G^+^ and CD11b^+^ cells, from the mice shown in (D) were assessed for their expression of CD11b. Representative FACS plots are shown. The percentage of CD11b^hi^ and CD11b^lo^ cells is indicated. Statistical significance of any differences for A, B and C was determined using a 1-way ANOVA with Tukey post-test analysis. Statistical significance of any differences for D was determined by 2-tailed *t* test. NS, not significant.

To determine if reduced GM-CSF production by NK cells and impaired neutrophil microbicidal activity are linked, we investigated if NK cells from control mice could restore the resistance of CD11cΔSyk mice to infection. Transfer of cell preparations enriched for NK cells from naïve control mice into CD11cΔSyk mice prior to infection had no impact on fungal burden (Figure 5A). However, when the same preparations were isolated from infected control mice, fungal control was restored in subsequently infected CD11cΔSyk mice (Figure 5A). The decrease in fungal burden conferred by adoptive NK cell transfer associated with an increased proportion of CD11b^hi^ neutrophils (Figure 5B) and restoration of the ability of neutrophils to kill *C. albicans ex vivo* (Figure 5C). Similar results were obtained upon transfer of pure NK cell populations that were isolated by cell sorting to exclude any confounding effect of contaminants (Figure 5D, 5E). Protection was not observed when NK cells were isolated from mice infected 48 h earlier (Figure 5D and data not shown), consistent with the notion that GM-CSF production is transient (see above). Transfer of unsorted total spleen cells was also not protective even when the cells were taken at 16 h after infection (Figure 5D, 5E and data not shown). We conclude that the susceptibility of CD11cΔSyk mice to *C. albicans* infection is due to a defect in GM-CSF-dependent NK cell “help” for neutrophils and can be prevented by transfer of appropriately-primed NK cells from recently-infected wild type mice but not by unprimed wild-type NK cells.

### IL-23p19 links Syk signaling in CD11c^+^ cells to GM-CSF-production by NK cells

**Figure 6:**
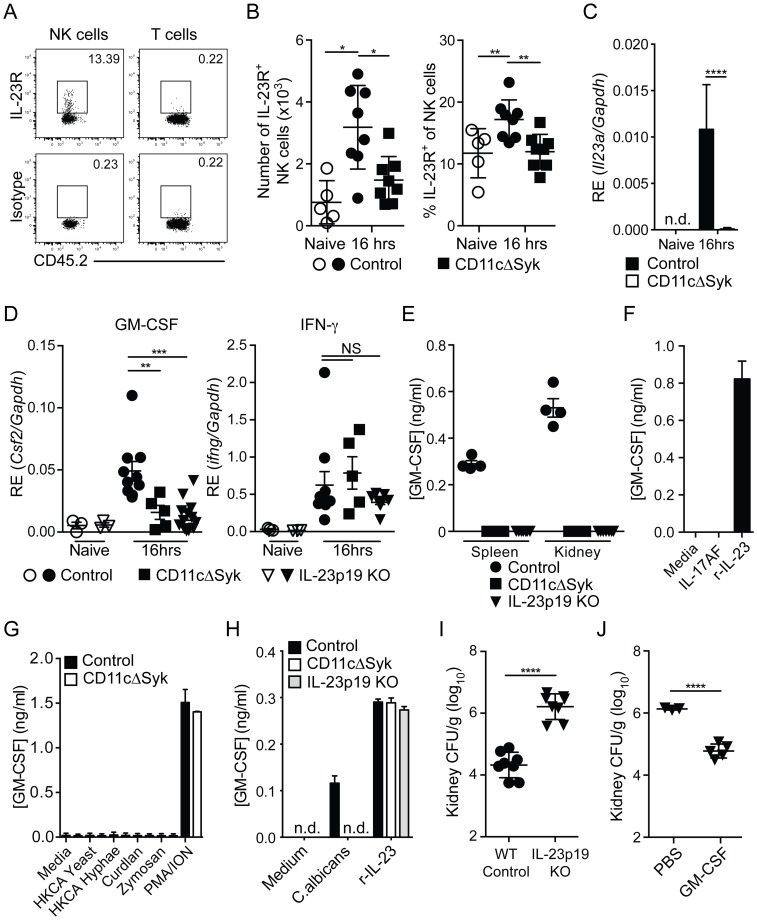
Defect in IL-23p19 production by Syk-deficient kidney DCs underlies decreased NK cell GM-CSF-mediated control of *C. albicans*. (A) Representative staining of naïve control kidney NK cells (CD45.2^+^ NK1.1^+^ CD3^−^) and T cells (CD45.2^+^ CD3^+^) with anti-IL-23R vs. isotype-matched irrelevant specificity control (B) Percentage and total number of kidney NK cells expressing IL-23R in control and CD11cΔSyk mice that were either uninfected or infected 16 h earlier. Data are mean +/− SEM from three independent experiments with each symbol representing an individual mouse. (C) CD11c^+^ MHC-II^+^ cells were purified by cell sorting from the kidneys of naïve or 16 h post-infection control and CD11cΔSyk mice. RNA was extracted and qRT-PCR performed to detect *il23a transcripts*. Data shown are mean +/− SEM from two independent experiments with five biological replicates. n.d., not detected. (D) NK cells were purified by cell sorting from the kidneys of naïve and 16 h post-infection control, CD11cΔSyk and IL-23p19 KO mice. RNA was extracted and qRT-PCR performed to detect levels of *Csf2* and *Ifng* transcripts. Data shown are mean +/− SEM from two independent experiments with each symbol representing an individual mouse. (E) NK cells were sorted from the spleen and kidney of 16 h-infected control, CD11cΔSyk and IL-23p19KO mice. Cells were cultured overnight and supernatants were collected and assessed for GM-CSF protein content by ELISA. Data shown are mean +/− SEM from two independent experiments with each symbol representing data from an individual animal. (F) Splenic NK cells were sorted from naïve control mice and stimulated with medium, recombinant IL-17AF heterodimer or recombinant IL-23 (r-IL-23) overnight. GM-CSF protein in the supernatants was measured by ELISA. Data shown are mean +/− SEM of triplicate wells from one experiment. (G) Splenic NK cells were sorted from naïve control and CD11cΔSyk mice and were stimulated overnight with medium, heat-killed *C. albicans*, Curdlan, Zymosan or PMA/Ionomycin. GM-CSF accumulation in the supernatants was assessed by ELISA. Data shown are mean +/− SEM of duplicate wells from one out of two independent experiments. (H) Splenic NK cells were sorted from naïve control mice and co-cultured with BMDCs derived from control, CD11cΔSyk or IL-23p19KO mice. Co-cultures were stimulated with medium, heat-killed *C. albicans* or recombinant IL-23 (r-IL-23) overnight and GM-CSF protein accumulation in the supernatants was assessed by ELISA. Data shown are mean +/− SEM of triplicate wells from one out of two independent experiments. n.d., not detected. (I) Control and IL-23p19 KO mice were infected with 2×10^5^ CFU of *C. albicans* intravenously. Kidneys were removed 2 days post-infection and analyzed for fungal burden. Data are mean +/− SEM from two independent experiments with each symbol representing an individual animal. (J) IL-23p19 KO mice were given PBS or GM-CSF at the time of *C. albicans* infection and a second dose 24 h later. Fungal burden was assessed 2 days post-infection. Data are mean +/− SEM pooled from two independent experiments with each symbol representing an individual animal. Statistical significance of any differences for B was determined using a 1-way ANOVA with Tukey post-test analysis. C, I and J were assessed using a 2-tailed *t* test Whilst a Kruskal-Wallis with Dunn's multiple comparison test was undertaken for D. NS, not significant.

Finally, we sought to identify the signal that links Syk signaling in DCs to the production of GM-CSF by kidney NK cells. IL-23p19 has been reported to be induced very rapidly yet transiently in the kidneys and lungs of *Candida*-infected mice [53], [54]. In addition, IL-23p19 is important for early resistance to candidiasis [55], [56] and can be synthesized by DCs in a Syk-dependent manner upon stimulation with CLR agonists [27]. Although IL-23R can be found on both NK cells and T cells [57], we noted that NK cells but not T cells expressed IL-23R in the kidney (Figure 6A). The expression of IL-23R on kidney NK cells was upregulated in control but not CD11cΔSyk mice following infection (Figure 6B). In addition, we detected strong induction of IL-23p19 mRNA in kidney CD11c^+^ MHC-II^+^ DCs from control mice but, importantly, not from CD11cΔSyk mice early after infection with *C. albicans* (Figure 6C). The increase in the proportion of IL-23R^+^ cells (Figure 6B) may therefore be a consequence of positive feedback signaling of IL-23R in response to ligand [57], [58].

To test the significance of this observation, we infected IL-23p19 KO mice and measured NK cell production of GM-CSF. Notably, IL-23p19-deficient mice resembled CD11cΔSyk mice in that NK cells taken from the kidneys of either strain displayed markedly reduced levels of GM-CSF but not IFN-γ mRNA and did not secrete GM-CSF protein upon short-term *ex vivo* culture (Figure 6D and 6E). Furthermore, purified NK cells produced GM-CSF *in vitro* when stimulated with recombinant IL-23 but not IL-17A/F, *C. albicans*, curdlan or zymosan (Figure 6F and 6G). GM-CSF production by NKs in response to *C. albicans* occurred only in the presence of DCs derived from wild-type but not CD11cΔSyk or IL-23p19-deficient mice (Figure 6H). Finally, IL-23p19 KO mice infected with *C. albicans* were undistinguishable from CD11cΔSyk mice in having massively increased kidney fungal burdens (Figure 6I) that could be reversed by GM-CSF therapy (Figure 6J). Together, these data suggest that Syk-dependent IL-23p19 production by DCs in response to *C. albicans* acts directly on NK cells to promote GM-CSF production and subsequent resistance to systemic candidiasis.

## Discussion

Multiple receptors on macrophages, monocytes, neutrophils, NK cells and innate lymphocytes, as well as on non-immune cells, mediate the recognition of microbes and are thought to act co-ordinately and somewhat redundantly to provide innate resistance to infection. Here, we demonstrate that DCs, a cell type chiefly known for its ability to initiate adaptive immunity, coordinate the entire innate immune control to systemic infection with *C. albicans* and show that this orchestration depends on a single kinase, indicating a remarkable lack of redundancy in innate immune pathways. We further unravel a hitherto unappreciated series of cellular interactions whereby DCs provide IL-23p19 to NK cells that allows for production of GM-CSF, which in turn maintains the microbicidal activity of neutrophils, the main candidacidal effectors. Disruption of this cellular relay in CD11cΔSyk or IL-23p19 KO mice causes susceptibility to systemic candidiasis and restoration of resistance can be achieved with GM-CSF treatment. Thus, our analysis reveals Syk mediated IL-23p19 production by DCs as a central and non-redundant node of innate immunity to fungal infection and an unexpected indirect regulator of neutrophil microbicidal activity via NK cells.

Although the central function of neutrophils in innate protection from disseminated candidiasis is undisputed, the role of mononuclear phagocyte populations is not well established. It is surprising that loss of Syk from CD11c^+^ cells in CD11cΔSyk mice causes such a dramatic phenotype. We show that this is not because Syk uniquely regulates the development of particular CD11c^+^ subsets that coordinate anti-fungal immunity or even their recruitment to the site of infection, as there were no gross alterations in the composition of CD11c^+^ populations in kidneys from infected CD11cΔSyk mice. As in spleen and many other organs, kidney CD11c^+^ cells are also MHC-II^+^ and would therefore traditionally be defined as DC. However, not all kidney CD11c^+^ MHC-II^+^ cells are derived from committed DC precursors, leading to debate as to whether they are best classified as DCs or macrophages [59], [60]. Taking advantage of a new Clec9a-Cre line to selectively target those cells derived from pre-DC/CDP [44], we show that deletion of Syk in the DC lineage (as defined hematopoietically) phenocopies deletion in total CD11c^+^ cells. This would suggest that the susceptibility of CD11cΔSyk mice to systemic candidiasis is primarily due to loss of Syk from DCs. This in turn adds to the emerging notion that DCs may act as central regulators of innate immunity to infection in some instances [61]. Loss of resistance to *Candida* was also seen in LysMΔSyk mice (data not shown) and we do not presently exclude a possible contribution of Syk on CD11c^+^ cells of monocytic origin (although we note that such a result is ambiguous as LysM-Cre activity is also found on conventional non-monocytic DCs [62]). Whichever their origin, the central role of Syk in DCs suggests that ablation of CD11c^+^ cells should also have a dramatic phenotype on resistance to *Candida* infection. Surprisingly, this was not the case as CD11c-DTR (diphtheria toxin receptor) mice treated with diphtheria toxin were actually more resistant to infection with *C. albicans* (data not shown). This apparent discrepancy can be explained by the recent observation that ablation of CD11c^+^ cells in CD11c-DTR mice is accompanied by marked neutrophilia, which provides a major barrier to bacterial or, in this case, fungal infection [63].

It is notable that deletion of MyD88 in CD11c^+^ cells had no impact on *C. albicans* infection even though it markedly impacts responses to TLR agonists *in vivo* [41]. This may suggest a primacy of Syk-coupled rather than MyD88-coupled receptors in fungal recognition by DCs [1], [22], [64]. Nevertheless, MyD88 remains an important component of anti-fungal resistance as we find, along with Villamon *et. al.* [65], that MyD88-deficient animals are very susceptible to systemic *C. albicans* infection. Unlike that of CD11cΔSyk mice, this susceptibility is not preventable by exogenous GM-CSF therapy and presumably involves MyD88 signaling in CD11c^−^ cells. Whether this happens downstream of TLRs or receptors for IL-1 family cytokines remains to be determined.

Depletion of neutrophils dramatically increases susceptibility of mice to experimental systemic *C. albicans* infection [31] and neutropenia places patients at severe risk from systemic candidiasis [32], [66]. Previous work showed that IL-6-deficient mice are highly susceptible to systemic *C. albicans* [67], which was attributed to a lack of neutrophil recruitment without impairment of candidacidal capacity [68]. It was therefore surprising to observe the opposite phenotype, namely normal neutrophil recruitment but impaired activity in infected CD11cΔSyk or Clec9aΔSyk mice. The ample production of neutrophil recruiting proteins such as IL-6, KC and MIP-2 (CXCL2) in the kidneys of such mice might account for unabated neutrophil recruitment. In contrast, the lack of GM-CSF in the microenvironment appears to be responsible for the loss of neutrophil activity. Neutrophil activation triggers re-localization of intracellular pools of CD11b to the plasma membrane [69] allowing for adhesion, migration and phagocytosis [48]. Thus, we suggest that the presence of CD11b^lo^ neutrophils is indicative of poorly activated cells with decreased microbicidal potential, as highlighted by our killing assays. Interestingly, GM-CSF has been linked to neutrophil functionality and survival via physical coupling of Src family kinase Lyn to the GM-CSF receptor, resulting in down-regulation of pro-apoptotic factors and up-regulation of anti-apoptotic pathways such as PI3K/Ark [70]–[72]. While we have failed to observe obvious signs of neutrophil apoptosis in CD11cΔSyk mice, we cannot exclude that any apoptotic cells might be removed rapidly and that the CD11b^lo^ phenotype is indeed a prelude to cell death. Notably, intravenous GM-CSF infusion is curative in cases of severe drug-resistant chronic mucocutaneous candidiasis [73] and patients with oral pseudomembranous candidiasis resulting from radiotherapy for head and neck cancers have been successfully treated with a GM-CSF mouthwash [74]. In addition, human neutrophil activation and survival relies in part on NK cell-derived cytokines, including GM-CSF [75], and activated human NK cells enhance neutrophil survival and promote an increase in neutrophil CD11b expression and ROS production in a GM-CSF dependent manner [76]. Thus, GM-CSF, in part derived from NK cells, may underlie resistance to *Candida* infection not only in mice but also in Man. This is seemingly at odds with the fact that NK cell deficiency is associated primarily with viral rather than fungal infections [77]. However, the very few NK cell-deficient individuals studied so far may not have been exposed to the conditions predisposing to systemic candidiasis such as catheter insertion or deep tissue surgery. Alternatively, NK-cell independent mechanisms may compensate in these individuals for GM-CSF-dependent fungal control. In this regard, the requirement for NK cells in antifungal immunity even in mice may vary depending on the *Candida* strain in question [34], [78].

We have recently shown that the functional development of NK cells in mice requires cell-intrinsic IL-17RA-mediated signals [37]. NK cells that develop in the absence of such signals are impaired in their ability to produce IFN-γ, kill target cells, as well as produce GM-CSF to control *Candida* infection [37]. Here, we show that even in IL-17RA-sufficient mice, where NK cell functional development is unaffected, the response of NK cells to acute *Candida* challenge is under stringent environmental control and requires exogenous priming signals. Priming signals for GM-CSF but not IFN-γ production in turn require Syk signaling in DCs as demonstrated by the fact that transfer of resting NK cells does not restore resistance of CD11cΔSyk mice to *Candida*, yet resistance is achieved if the transfer involves activated NK cells that were primed in an environment in which DCs express Syk. Together with the fact that Clec9aΔSyk and CD11cΔSyk phenocopy each other, this argues against the possibility that the defect in CD11cΔSyk mice is due to deletion of Syk in the NK cells themselves (even if a small population of NK cells can express CD11c and show evidence of Cre activity in CD11c-Cre mice [79]). Supporting this contention, purified NK cells do not respond directly to *C. albicans ex vivo* but will readily do so in the presence of DCs or conditioned medium from *Candida*-treated DC cultures. This is consistent with the notion that accessory cells, such as DCs, monocytes, and macrophages are necessary for activation of NK cells in response to most pathogens (reviewed in [80], [81]). Nevertheless, it is possible that the anti-fungal activity of primed NK cells additionally requires signaling via Syk-coupled NK cell receptors and it will be interesting to study the phenotype of mice in which Syk is selectively ablated in NK cells as opposed to DCs.

**Figure 7:**
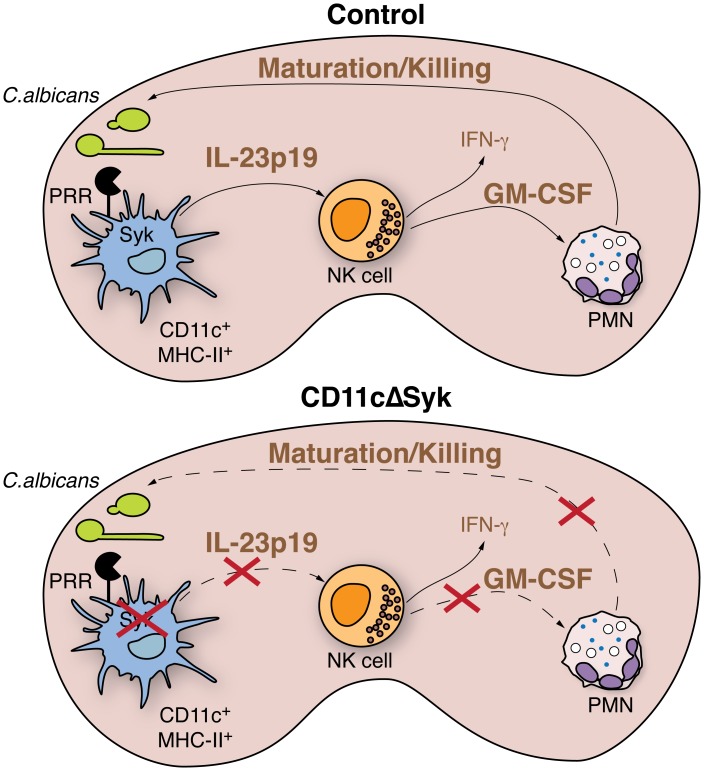
Model for Syk-mediated recognition of fungal particles by DCs in control of systemic candidiasis. Upon systemic infection, recognition of *C. albicans* by kidney DCs via Syk-coupled PRRs leads to production of IL-23p19. DC-derived IL-23p19 acts on NK cells, causing them to produce GM-CSF, which in turn acts on neutrophils infiltrating the kidney to promote and sustain fungicidal activity. Loss of Syk in DCs prevents production of IL-23p19 leading to failure of NK cells to provide GM-CSF-dependent help to neutrophils. Impaired killing capacity of the latter leads to loss of control of fungal burden.

It is well known that stimulation of DCs and macrophages by *C.albicans* yeast and hyphae induces the production of IL-2, IL-6, IL-12, IL-23 and TNF-α in a Syk-dependent manner [21], [27]. In searching for which one of these or other factors might be responsible for priming NK cells to produce GM-CSF we focused on IL-23p19. We show that IL-23p19 is not induced in Syk-deficient DCs during systemic candidiasis and that *Candida*-stimulated control but not IL-23p19 KO DCs induce GM-CSF production by NK cells. We further show that IL-23p19 KO mice are very susceptible to systemic candidiasis, as previously suggested [55], but can be protected by GM-CSF treatment. Together, these data suggest that IL-23 might be the key Syk-dependent cytokine driver of DC-mediated resistance to candidiasis, consistent with the fact that addition of recombinant IL-23 and not recombinant IL-17A/F to purified NK cells induces GM-CSF production. This sheds light on a novel regulatory mechanism of cytokine production by NK cells that selectively affects GM-CSF but not IFN-γ secretion. However, IL-23 is composed of both the IL-23p19 and the IL-12/IL-23p40 subunits and it has been reported that IL-12p40-deficient mice are resistant to candidiasis [82], [83]. We have been able to reproduce this finding (unpublished observations) and therefore, at present, we are forced to conclude that the key mediator of resistance is either a novel IL-23p19-containing cytokine (including, possibly, an IL-23p19 homodimer) or that IL-23 deficiency impairs resistance to candidiasis in an IL-12-sufficient but not IL-12-deficient background. While work to assess these possibilities is ongoing, our existing data nevertheless argue for a model (Figure 7) where Syk-mediated recognition of fungal particles by DC, possibly through Syk-coupled CLRs, leads to production of an IL-23p19-containing cytokine, which acts on NK cells in the kidney to induce GM-CSF production. In turn, GM-CSF acts on recruited neutrophils to sustain microbicidal function. This unusual cellular relay from DCs to NK cells and to neutrophils via IL-23p19 and GM-CSF, respectively, provides a key axis for protection from disseminated candidiasis in mice that may be worth exploring as a possible therapeutic target in the context of fungal sepsis in humans.

## Materials and Methods

### Ethics statement

All animal protocols were carried out under the authority of a UK project license (number PPL 80/2309) approved by the CRUK London Research Institute Animal Welfare and Ethical Review Body in strict accordance with UK governmental regulations (Animal Scientific Procedures Act 1986) or under protocols approved by the Veterinary office of the Canton Zürich, Switzerland (license number 184/2009 and 201/2012) in strict accordance with the guidelines of the Swiss Animal Protection Law. All efforts were made to minimize animal suffering and ensure the highest ethical and humane standards.

### Mice

Control mice ‘Control’ (including C57Bl/6 and littermate CD11cCre^−^ [42]×Syk^flox/flox^ [40]), CD11cΔSyk (CD11cCre^+^×Syk^flox/flox^), CD11cΔMyD88 (CD11cCre^+^×MyD88^flox/flox^ [41]), Control MyD88 (CD11cCre^−^×MyD88^flox/flox^), MyD88 KO [84], Clec9a(egfp/egfp) [45] (DNGR1 deficient), Clec9aΔSyk (Clec9a^Cre/Cre^ [44]×Syk^flox/flox^), Clec9a control (Clec9a^+/+^×Syk^flox/flox^) and IL-23p19 KO [85], [86] were crossed and bred at Cancer Research UK and at the Institute of Laboratory Animal Sciences, University of Zürich, Switzerland, in specific pathogen-free conditions.

### Cells and fungal stimuli

*Candida albicans* strains SC5314 and CAI4-pACT1 GFP (described in [87]) were grown by agitation overnight at 30°C in yeast peptone dextrose (YPD) or synthetic complete medium (SC) containing 2% glucose and Drop-out mix without Uridine. The cells were then washed twice with PBS before use as live yeasts. Heat-killed *C. albicans* (HKCA) yeast or hyphae were generated by boiling samples for 1 h.

### Systemic *C. albicans* infection model, GM-CSF treatment and fungal counts

Mice aged 8–20 weeks were infected intravenously with 2×10^5^ live *C. albicans* yeast unless stated. The mice were killed 2 days post-infection except where indicated and perfused with cold PBS. Mice that received GM-CSF treatment had two intraperitoneal doses of murine GM-CSF (Peprotech) 5 µg/mouse at time of infection and 24 h later. The kidneys were removed and homogenized in 1 ml PBS using an IKA T25 digital Ultra-Turrax homogenizer or a Qiagen Tissue Lyser. Serial dilutions were plated on YPD agar plates and the total number of colony forming units was calculated.

### Histology

Samples were fixed in 10% Neutral Buffered Formalin and processed by the histopathology laboratory at Cancer Research UK. Samples were dehydrated with ethanol and embedded in paraffin. Periodic Acid Schiff (PAS) and hematoxylin and eosin (H&E) were used to assess fungal invasion and leukocyte infiltration respectively.

### Cell isolation

Single-cell kidney, spleen and bone marrow (BM) suspensions were prepared from PBS perfused mice. Kidneys and spleens were chopped into small pieces and digested in RPMI 1640 medium supplemented with glutamine, penicillin, streptomycin, (all from Gibco), collagenase type IV (200 u/ml, Worthington), and DNase 1 (0.2 mg/ml, Roche) for 1 h or 30 min respectively at 37°C. Cells were then passed through a 70 µm cell strainer (BD bioscience), washed with RPMI 1640 supplemented with 10% fetal calf serum, glutamine, penicillin and streptomycin (RPMI complete medium). Single cell kidney samples were then placed onto a non-continuous isotonic Percoll (GE Healthcare) gradient of 78% and 37% and centrifuged for 30 min at 550 g. The interface was collected from these samples and washed in RPMI complete medium. For isolation of BM cells, the femur and tibia were collected from both hindquarters. Bones were flushed with RPMI complete medium and passed through a 70 µm cell strainer to obtain single cell suspensions. Splenic and BM erythrocytes were lysed with Red Blood Cell Lysis Buffer (Sigma) for 3 min at room temperature (RT). Single cell populations were subsequently used for either FACS staining, *in vitro* candidacidal activity or cell sorting.

### Flow cytometry and cell staining and intracellular cytokine staining

Data were collected on LSR Fortessa, FACSAria or LSRII (all BD Biosciences) and analyzed using FlowJo software (Tree Star). The staining protocols used combinations of antibodies listed below. Antibodies purchased from BD bioscience included: anti-CD3e (145-2C11), anti-CD4 (RM4-5), anti-CD8 (53-6.7), anti-CD11b (M1/70), anti-CD11c (HL3), anti-CD16/CD32 (2.4G2, Fc block), anti-CD19 (1D3), anti-CD45R/B220 (RA3-6B2), anti-CD49b (DX5), anti-CD64 (X54-5/7.1), anti-IFN-γ (XMG1.2), anti-Ly6G (1A8) and Streptavidin-APC. The following antibodies were purchased from eBioscience: anti-CD3e (145-2C11), anti-CD11b (M1/70), anti-CD103 (2E7), anti-GM-CSF (MP1-22E9), anti-MHC-II (M5/114.15.2) and anti-NK1.1 (PK136). The following antibodies were purchased from Biolegend: anti-CD11a (M17/4), anti-CD11c (N418), anti-CD18 (M18/2), anti-CD45.2 (104), anti-F4/80 (BM8), anti-Syk (5F3, purified and conjugated to AF647 with AF647 Antibody labelling kit (Molecular Probes)). Additional antibodies used were Polyclonal Goat anti-Mouse/Rat MyD88 (R&D), anti-Mouse IL-23R (753317, R&D), Rabbit anti-Goat AF488 (Molecular Probes) and anti-MPO (8F4, biotinylated, Hycult Biotech).

Single cell suspensions were surface stained directly *ex vivo* or following 7 h incubation with brefeldin A (Sigma). Most cell staining involved dead cell exclusion by live/dead fixable violet dye (Invitrogen), followed by fixation with 2% paraformaldehyde (Electron Microscopy Sciences) for 20 min at RT. Cells were washed twice with FACS buffer (PBS, 1% FCS, 2 mM EDTA). For intracellular staining, cells were subsequently permeabilised and stained with saponin containing Reagent B (ADG Bio Research GMBH).

### Measurement of chemokine and cytokine protein levels

Kidneys were removed 1 or 2 days post-infection following PBS perfusion and homogenized on ice in 0.5 or 1 ml of PBS respectively. Chemokines and cytokines from homogenates and cell culture supernatants were analyzed according to manufacturer's instructions. Briefly, clarified samples were incubated with either BD cytometric bead array kits (IL-6, KC, MIP-1α, TNF, IL-1α), FlowCytomix Kits (IL-15/IL-15R, MCP-3 and IL-10), R&D Quantikine ELISA kit (IL-1β) or eBioscience Ready-Set-Go ELISA kit (GM-CSF). Bead based assays were assessed using a LSR Fortessa whilst ELISA samples were read at 450 nm with all concentrations determined relative to a standard curve.

For proteome profiling, kidneys were removed from naïve or 16 h post-infection mice following PBS perfusion and homogenized in 1 ml PBS with protease inhibitor cocktail (cOmplete Roche) with Triton ×100 added at a final concentration of 1% prior to a freeze thaw step. Samples were clarified prior to addition to the R&D Proteome profiler (Mouse cytokine array panel A) and developed as per manufacturer's instructions. Relative pixel density of each duplicate blot was measured using Image J software. The data are presented as fold change in signal from infected samples compared to naïve control samples.

### Neutrophil isolation and functional assay

Single cell suspensions of kidney and bone marrow were prepared as above prior to staining and sorting for neutrophils, identified by CD11b^+^ Ly-6G^+^ F4/80^−^ and GFP^+^ or GFP^−^. Sorted neutrophils were incubated with *C. albicans* (10∶1) in serum free medium on ultra low attachment plates (Costar) for 1 h at 37°C. Wells were collected and cells lysed with water prior to plating on YPD agar. *C. albicans* colony formation from neutrophil-containing wells was compared to that from control neutrophil-free wells to calculate the percentage of *C. albicans* killed. Data are combined from three independent experiments with each data point representing an individual well. Neutrophils were also isolated from mouse blood using a density gradient of Histopaque 1119 and Histopaque 1077 (both Sigma). Blood neutrophil killing activity was assessed using 10^4^ *C. albicans* yeast co-cultured with 10^4^ neutrophils (usually >80% Ly6G^+^) in protein low binding tubes (Sarstedt) for 2 h. The percentage of *C. albicans* killed was assessed as above with data combined from two independent experiments.

### NK cell purification and transfer

Single cell suspensions of spleens from control mice were obtained as described above and NK cells were either enriched with anti-DX5 microbeads (Miltenyi Biotech) or purified by FACS based on DX5 and CD3 expression. 8×10^6^ enriched NK cells or 4×10^6^ FACS purified NK cells (>95% pure and viable) were adoptively transferred into recipient mice 1 h prior to infection.

### NK cell—DC co-cultures

DCs were differentiated from BM precursors in presence of GM-CSF for 7 days. 10^5^ FACS-purified NK cells from naïve spleens were cultured alone or co-cultured with 5×10^4^ DCs in presence of heat-killed *C. albicans* (10 M.O.I.), 100 µg/ml Curdlan, 50 µg/ml Zymosan, recombinant IL-23 (BD bioscience; 100 ng/ml) or recombinant IL-17A/F heterodimer (BD bioscience; 1 µg/ml). The culture supernatant was collected after overnight incubation and GM-CSF was quantified by ELISA (eBioscience) according to manufacturer's instructions.

### Quantitative RT-PCR

RNA was extracted from whole organs disrupted using the Tissue Ruptor (Qiagen) using TRIzol (Invitrogen) according to manufacturer's instructions. RNA from FACS sorted cell samples was isolated using either TRIzol or the QIAcube (Qiagen). Isolated RNA was reverse transcribed into complementary DNA using random primers (Invitrogen). Quantitative PCR was performed using Taqman primer/probe sets (Invitrogen), Sykb (Mm01333035_m1 (exon boundary 1–2)), *csf2* (Mm00438328_m1), *ifng* (Mm01168134_m1) and house keeping Gapdh (Mm99999915_g1) or SYBR Green (Qiagen) with primer pairs *il23a* (F-GCCAAGAAGACCATTCCCGA R-TCAGTGCTACAATCTTCTTCAGAGGACA) and Gapdh (F-CAGTATTCCACTCTGAAGAAC R-ATACGGCCAAATCTGAAAGAC) using either the Viia7 or 7500 Fast Real-Time PCR System (Applied biosystems).

### Statistical analysis

Prism version 6a (GraphPad) was used for plotting data and for statistical analysis. Survival data are presented as a Kaplan-Meier plot with a log rank test used to compare significance between groups. Data was subjected to D'Agostino & Pearson omnibus normality test, Shapiro-Wilk normality test and Kolmogorov-Smirnov test to determine the subsequent statistical tests applied. Statistical significance of differences between two groups or groups with fewer than three samples was determined by 2-tailed *t* test. For experiments with more than 2 groups, significance of any differences was determined using a 1-way ANOVA with Tukey multiple comparison of all pairs for post-test analysis. If the data was assessed to be non-gaussian then a Kruskal-Wallis with Dunn's multiple comparison test was undertaken. The level of significance was defined as *p<0.05, ** p<0.01, *** p<0.001, **** p<0.0001. The test used for statistical analysis is indicated in each figure legend.

## Supporting Information

Figure S1 **Expression of Syk and MyD88 in the kidney and spleen of naïve and infected mice.** Kidneys and spleens were removed from either naïve or 2 day infected mice following PBS perfusion. Organs were treated with collagenase IV/DNase I prior to enrichment using a 37%–78% percoll gradient. Leukocytes were surface stained with CD45.2 and then identified as B cells (CD19^+^, MHC-II^+^), T cells (CD3^+^ CD4^+^ or CD8^+^), neutrophils (Ly-6G^+^ CD11b^+^) and CD11c^+^ MHC-II^+^ (subset with CD11b, F4/80 and CD103). (A) *syk* mRNA levels were measured by qRT-PCR from kidney and spleen sorted cell populations. Data shown are mean +/− SEM from two independent sorts with six biological samples with statistical significance of any differences determined by 2-tailed *t* test. (B) Naïve samples were permeabilised and stained with anti-Syk and analysed for Syk expression. Representative Syk expression in CD11c^+^ MHC-II^+^, neutrophils and B cells from naïve mice. (C) Geometric mean of Syk expression by the indicated leukocyte populations from naïve kidneys. Data shown are mean +/− SEM from one representative experiment of two with statistical significance of any differences determined by 2-tailed *t* test. (D) Samples were permeabilised and stained with anti-MyD88 with a rabbit anti-goat IgG AF488 secondary and analysed for MyD88 expression. Histograms show MyD88 expression for CD11c^+^ MHC-II^+^ cells and neutrophils from naïve mice with each line representing an individual mouse. (E) Representative Syk expression by the indicated subpopulations of kidney CD11c^+^ MHC-II^+^ cells from day 2 infected kidneys.
(TIF)

Figure S2 **Mononuclear leukocyte subset composition is unaltered in the kidneys of CD11cΔSyk mice.** Control and CD11cΔSyk mice were infected with 2×10^5^ CFU of *C. albicans* intravenously. Kidneys were removed from naïve and 2 days infected mice and leukocyte populations were identified following surface staining for CD45.2, CD11c, CD11b, F4/80 and MHC-II. (A) Percentage and total number of CD45.2^+^ CD11c^+^ MHC-II^+^ cells in the kidneys of naïve and day 2 infected mice. (B) Representative profiles after gating on CD45.2^+^ CD11c^+^ MHC-II^+^ cells. (C) Percentage and total number of cells within subpopulations of kidney CD11c^+^ MHC-II^+^ cells. Data shown in A and C are mean +/− SEM from 3 pooled experiments with each symbol representing an individual mouse with statistical significance of any differences determined by 2-tailed *t* test.
(TIF)

Figure S3 **Overall increase of inflammatory cytokines and chemokines in the kidney following infection.** Kidneys were removed 1 (A) or 2 (B) days post-infection following PBS perfusion and homogenized on ice in 0.5 or 1 ml of PBS respectively. Cytokines and chemokines in clarified supernatants were quantified by either BD cytometric bead array kits (IL-6, KC, MIP-1α, TNF-α, IL-1α), FlowCytomix Kits (IL-15/IL-15R, MCP-3 and IL-10) or R&D Quantikine ELISA kit (IL-1β). Data shown are mean +/− SEM from 4 pooled experiments with each symbol representing an individual mouse with statistical significance of any differences determined by 2-tailed *t* test. (C) Kidneys were removed from naïve or 16 h post-infection mice following PBS perfusion and homogenized in 1 ml PBS with protease inhibitor and Triton ×100 added to a final concentration of 1% prior to a freeze-thaw step. Samples were clarified prior to addition to the R&D Proteome profiler (Mouse cytokine array panel A) according to manufacturer's instructions. The relative pixel density of each duplicate blot was assessed using Image J software and compared between naïve and infected samples. Data shown is a selection of the total proteome analysis showing increased (left panel) similar (middle panel) decrease protein levels (right panel) in the CD11cΔSyk mice compared to control mice.
(TIF)
