# Correction: Syk Signaling in Dendritic Cells Orchestrates Innate Resistance to Systemic Fungal Infection

**DOI:** 10.1371/journal.ppat.1007366

**Published:** 2018-11-21

**Authors:** Paul G. Whitney, Eva Bär, Fabiola Osorio, Neil C. Rogers, Barbara U. Schraml, Safia Deddouche, Salomé LeibundGut-Landmann, Caetano Reis e Sousa

There is an error in the caption for Fig 6, “Defect in IL-23p19 production by Syk-deficient kidney DCs underlies decreased NK cell GM-CSF-mediated control of *C*. *albicans*,” panel F. Please see the complete, correct Fig 6 caption here.

**Fig 6:**
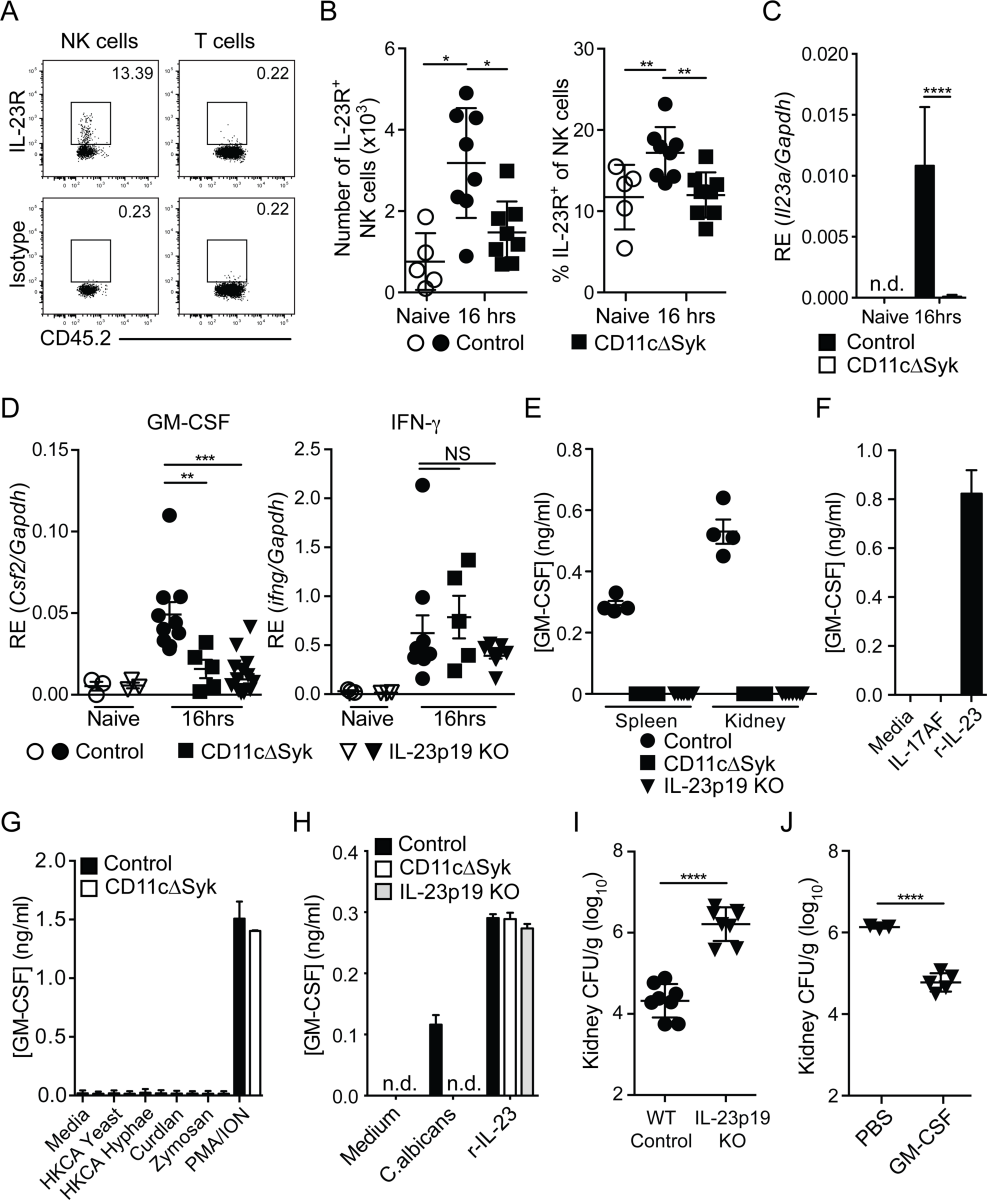
Defect in IL-23p19 production by Syk-deficient kidney DCs underlies decreased NK cell GM-CSF-mediated control of *C*. *albicans*. (A) Representative staining of naïve control kidney NK cells (CD45.2^+^ NK1.1^+^ CD3^−^) and T cells (CD45.2^+^ CD3^+^) with anti-IL-23R vs. isotype-matched irrelevant specificity control (B) Percentage and total number of kidney NK cells expressing IL-23R in control and CD11cΔSyk mice that were either uninfected or infected 16 h earlier. Data are mean +/− SEM from three independent experiments with each symbol representing an individual mouse. (C) CD11c^+^ MHC-II^+^ cells were purified by cell sorting from the kidneys of naïve or 16 h post-infection control and CD11cΔSyk mice. RNA was extracted and qRT-PCR performed to detect *il23a transcripts*. Data shown are mean +/− SEM from two independent experiments with five biological replicates. n.d., not detected. (D) NK cells were purified by cell sorting from the kidneys of naïve and 16 h post-infection control, CD11cΔSyk and IL-23p19 KO mice. RNA was extracted and qRT-PCR performed to detect levels of *Csf2* and *Ifng* transcripts. Data shown are mean +/− SEM from two independent experiments with each symbol representing an individual mouse. (E) NK cells were sorted from the spleen and kidney of 16 h-infected control, CD11cΔSyk and IL-23p19KO mice. Cells were cultured overnight and supernatants were collected and assessed for GM-CSF protein content by ELISA. Data shown are mean +/− SEM from two independent experiments with each symbol representing data from an individual animal. (F) NK cells were enriched from the spleen of naïve control mice and stimulated with medium, recombinant IL-17AF heterodimer or recombinant IL-23 (r-IL-23) overnight. GM-CSF protein in the supernatants was measured by ELISA. Data shown are mean +/- SEM of triplicate wells from one experiment. (G) Splenic NK cells were sorted from naïve control and CD11cΔSyk mice and were stimulated overnight with medium, heat-killed *C*. *albicans*, Curdlan, Zymosan or PMA/Ionomycin. GM-CSF accumulation in the supernatants was assessed by ELISA. Data shown are mean +/− SEM of duplicate wells from one out of two independent experiments. (H) Splenic NK cells were sorted from naïve control mice and co-cultured with BMDCs derived from control, CD11cΔSyk or IL-23p19KO mice. Co-cultures were stimulated with medium, heat-killed *C*. *albicans* or recombinant IL-23 (r-IL-23) overnight and GM-CSF protein accumulation in the supernatants was assessed by ELISA. Data shown are mean +/− SEM of triplicate wells from one out of two independent experiments. n.d., not detected. (I) Control and IL-23p19 KO mice were infected with 2×10^5^ CFU of *C*. *albicans* intravenously. Kidneys were removed 2 days post-infection and analyzed for fungal burden. Data are mean +/− SEM from two independent experiments with each symbol representing an individual animal. (J) IL-23p19 KO mice were given PBS or GM-CSF at the time of *C*. *albicans* infection and a second dose 24 h later. Fungal burden was assessed 2 days post-infection. Data are mean +/− SEM pooled from two independent experiments with each symbol representing an individual animal. Statistical significance of any differences for B was determined using a 1-way ANOVA with Tukey post-test analysis. C, I and J were assessed using a 2-tailed *t* test Whilst a Kruskal-Wallis with Dunn's multiple comparison test was undertaken for D. NS, not significant.
