# Supplementary figures and images for: Syk Signaling in Dendritic Cells Orchestrates Innate Resistance to Systemic Fungal Infection
Source: PLoS Pathog. 2014 Jul 17;10(7):e1004276. doi: 10.1371/journal.ppat.1004276 (PMC4102599; doi:10.1371/journal.ppat.1004276)

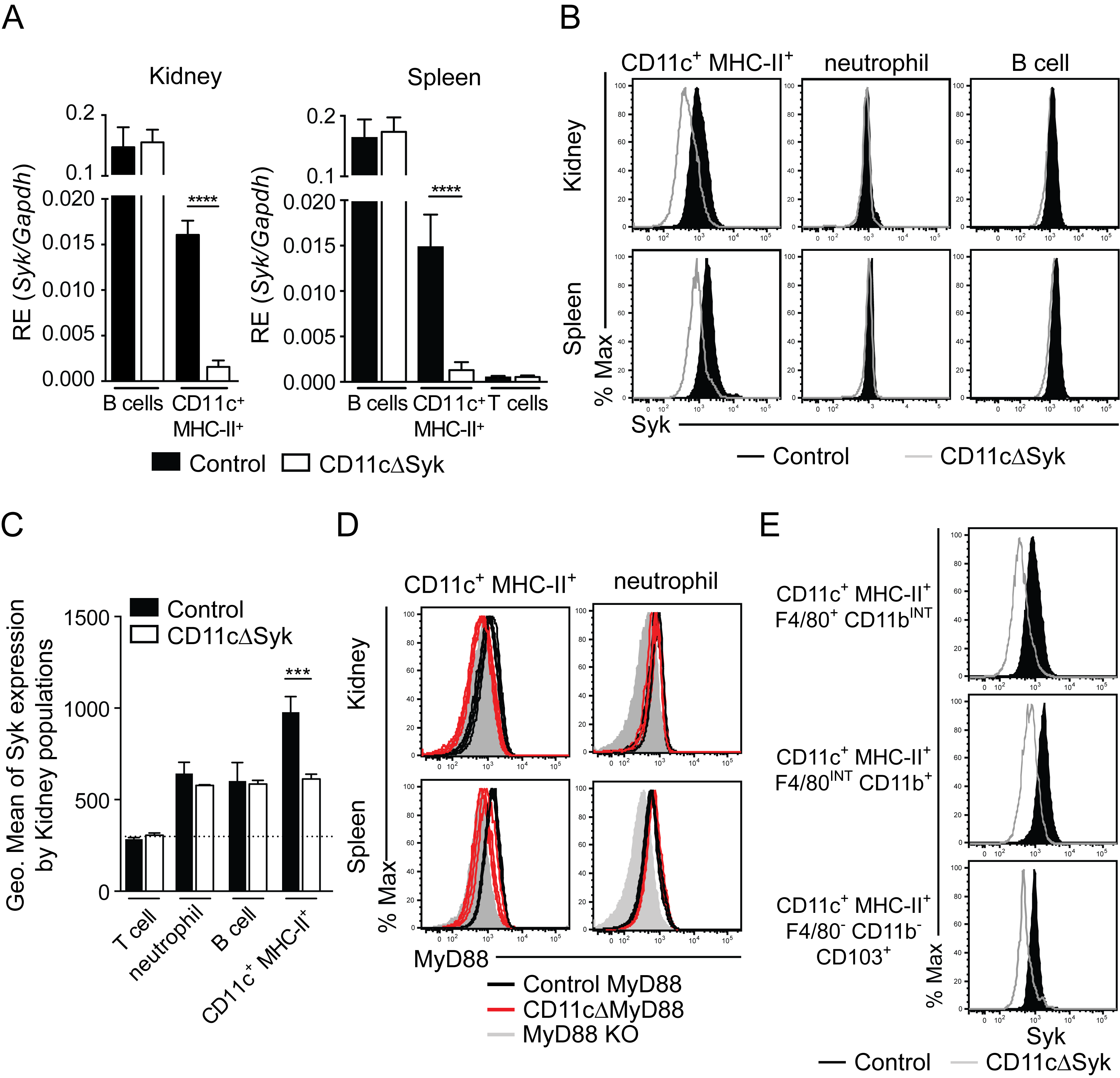

Supplement: Figure S1 — Expression of Syk and MyD88 in the kidney and spleen of naïve and infected mice. Kidneys and spleens were removed from either naïve or 2 day infected mice following PBS perfusion. Organs were treated with collagenase IV/DNase I prior to enrichment using a 37%–78% percoll gradient. Leukocytes were surface stained with CD45.2 and then identified as B cells (CD19+, MHC-II+), T cells (CD3+ CD4+ or CD8+), neutrophils (Ly-6G+ CD11b+) and CD11c+ MHC-II+ (subset with CD11b, F4/80 and CD103). (A) syk mRNA levels were measured by qRT-PCR from kidney and spleen sorted cell populations. Data shown are mean +/− SEM from two independent sorts with six biological samples with statistical significance of any differences determined by 2-tailed t test. (B) Naïve samples were permeabilised and stained with anti-Syk and analysed for Syk expression. Representative Syk expression in CD11c+ MHC-II+, neutrophils and B cells from naïve mice. (C) Geometric mean of Syk expression by the indicated leukocyte populations from naïve kidneys. Data shown are mean +/− SEM from one representative experiment of two with statistical significance of any differences determined by 2-tailed t test. (D) Samples were permeabilised and stained with anti-MyD88 with a rabbit anti-goat IgG AF488 secondary and analysed for MyD88 expression. Histograms show MyD88 expression for CD11c+ MHC-II+ cells and neutrophils from naïve mice with each line representing an individual mouse. (E) Representative Syk expression by the indicated subpopulations of kidney CD11c+ MHC-II+ cells from day 2 infected kidneys. (TIF) [file ppat.1004276.s001.tif]

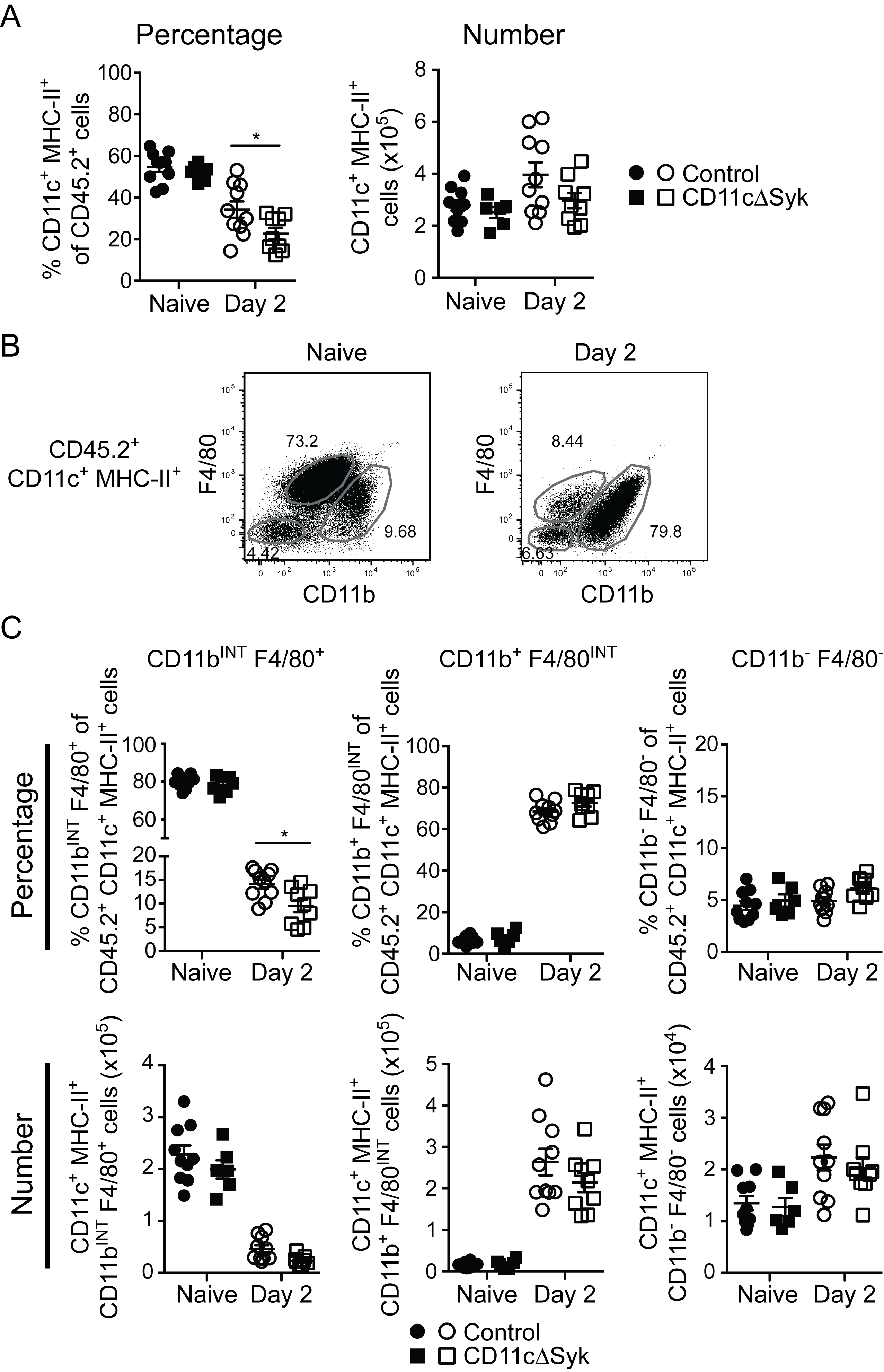

Supplement: Figure S2 — Mononuclear leukocyte subset composition is unaltered in the kidneys of CD11cΔSyk mice. Control and CD11cΔSyk mice were infected with 2×10^5^ CFU of C. albicans intravenously. Kidneys were removed from naïve and 2 days infected mice and leukocyte populations were identified following surface staining for CD45.2, CD11c, CD11b, F4/80 and MHC-II. (A) Percentage and total number of CD45.2+ CD11c+ MHC-II+ cells in the kidneys of naïve and day 2 infected mice. (B) Representative profiles after gating on CD45.2+ CD11c+ MHC-II+ cells. (C) Percentage and total number of cells within subpopulations of kidney CD11c+ MHC-II+ cells. Data shown in A and C are mean +/− SEM from 3 pooled experiments with each symbol representing an individual mouse with statistical significance of any differences determined by 2-tailed t test. (TIF) [file ppat.1004276.s002.tif]

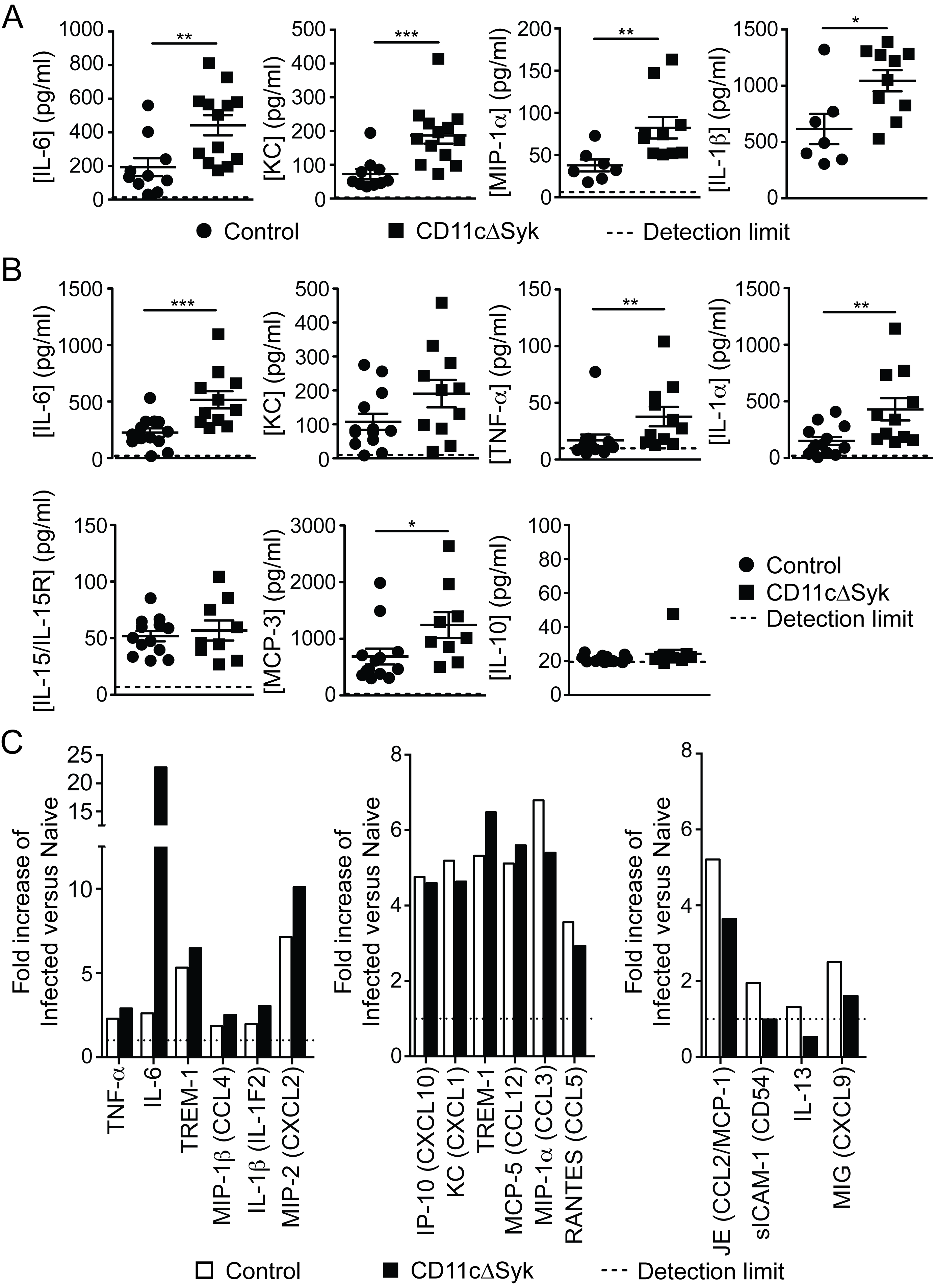

Supplement: Figure S3 — Overall increase of inflammatory cytokines and chemokines in the kidney following infection. Kidneys were removed 1 (A) or 2 (B) days post-infection following PBS perfusion and homogenized on ice in 0.5 or 1 ml of PBS respectively. Cytokines and chemokines in clarified supernatants were quantified by either BD cytometric bead array kits (IL-6, KC, MIP-1α, TNF-α, IL-1α), FlowCytomix Kits (IL-15/IL-15R, MCP-3 and IL-10) or R&D Quantikine ELISA kit (IL-1β). Data shown are mean +/− SEM from 4 pooled experiments with each symbol representing an individual mouse with statistical significance of any differences determined by 2-tailed t test. (C) Kidneys were removed from naïve or 16 h post-infection mice following PBS perfusion and homogenized in 1 ml PBS with protease inhibitor and Triton ×100 added to a final concentration of 1% prior to a freeze-thaw step. Samples were clarified prior to addition to the R&D Proteome profiler (Mouse cytokine array panel A) according to manufacturer's instructions. The relative pixel density of each duplicate blot was assessed using Image J software and compared between naïve and infected samples. Data shown is a selection of the total proteome analysis showing increased (left panel) similar (middle panel) decrease protein levels (right panel) in the CD11cΔSyk mice compared to control mice. (TIF) [file ppat.1004276.s003.tif]
